# Association between fatty acid compositions and genotypes of *FABP4 *and *LXR-alpha *in Japanese Black cattle

**DOI:** 10.1186/1471-2156-9-84

**Published:** 2008-12-11

**Authors:** Shogo Hoashi, Tomoko Hinenoya, Atsuko Tanaka, Hideki Ohsaki, Shinji Sasazaki, Masaaki Taniguchi, Kenji Oyama, Fumio Mukai, Hideyuki Mannen

**Affiliations:** 1 Laboratory of Animal Breeding & Genetics, Graduate School of Agricultural Science, Kobe University, Nada, Kobe 657-8501, Japan; 2 National Institute of Agrobiological Sciences, Tsukuba, Ibaraki 305-0901, Japan; 3 Food Resources Education & Research Center, Kobe University, Kasai, 675-2103, Japan

## Abstract

**Background:**

Fatty acid composition has become an important trait in the beef industry in terms of beef flavor and decreasing the circulating concentration of LDL cholesterol. In this study, we examined the association between polymorphisms of six genes, *adipocytes-type fatty acid binding protein *(*FABP4*), *liver X receptor α *(*LXRα*), *cytochrome b*_5 _(*Cyt b*_5_), *long-chain acyl-CoA synthetase *(*ACSL*) *1*, *ACSL4 *and *diacylglycerol acyltransferase 2 *(*DGAT2*) and fatty acid composition.

**Results:**

Sequence comparisons revealed 14 single nucleotide polymorphisms in six genes. Four of them, I74V and V110M in *FABP4 *and G51E and V133I in *LXRα*, were nonsynonymous substitutions. The associations between the genotypes and fatty acid compositions were analyzed by using 234 Japanese Black cattle. The genotypes of *FABP4 *I74V and *LXRα *V133I were significantly associated with palmitoleic acids (C16:1, P = 0.0086) and linoleic acid (C18:2, P = 0.0121) content in intramuscular fat, respectively.

**Conclusion:**

Our findings suggest that the two polymorphisms of *FABP4 *I74V and *LXRα *V133I might be genetic factors in part associated with palmitoleic acid (*FABP4 *I74V) and linoleic acid (*LXRα *V133I) composition in intramuscular fat of Japanese Black cattle, respectively. Especially, *FABP4 *I74V had highly significant effect (P < 0.01) on C16:1 proportion, indicating that the I/I homozygote exhibited 0.5% higher percentage than V/V homozygote.

## Background

Fatty acid composition of adipose tissue in beef cattle has become an important trait in the industry. In cattle adipose tissue, higher concentrations of monounsaturated fatty acid (MUFA) in the adipocytes and lower fat-melting point are considered to contribute to favorable beef flavor and may decrease the circulating concentration of LDL cholesterol [1-3]. The fatty acid composition in cattle, unlike that for non-ruminants, is much less dependent on the diet. Microorganisms within the rumen hydrogenate a majority of the dietary unsaturated fatty acids and most dietary fatty acids are absorbed as saturated fatty acids [4]. Zembayashi *et al*. [5] demonstrated that adipose tissue of Japanese Black cattle contains a higher proportion of MUFA than that of Holstein, Japanese Brown cattle or Charolais. These results suggested that fatty acid composition may be controlled by genetic factors such as lipid synthesis and fatty acid metabolism related genes.

*Stearoyl-CoA desaturase *(*SCD*) is a key enzyme responsible for conversion of saturated fatty acids (SFA) into MUFA. In our previous study, we demonstrated that a nucleotide substitution in the *SCD *coding sequence is associated with fatty acid composition in Japanese Black cattle [6]. In addition, we revealed that a 84 bp insertion in intron 5 of the *sterol regulatory element-binding protein-1 *(*SREBP-1*) affected the MUFA proportion in Japanese Black cattle [7]. SREBP-1 is a transcriptional factor that plays a pivotal role in energy homeostasis by promoting glycolysis, lipogenesis and adipogenesis. Zhang *et al*. [8] also demonstrated that the genotype of *fatty acid synthase *(*FASN*) was significantly associated with fatty acid composition of *Longissimus dorsi *muscle of Angus bulls. The results suggest that the fatty acid composition is controlled by polygenic factors.

The aim of this study is to discover additional genetic markers associated with fatty acid composition in beef. For this purpose, we picked six genes, *adipocytes fatty acid binding protein *(*FABP4*), *liver X receptor α *(*LXRα*), *cytochrome b*_5 _(*Cyt b*_5_), *long-chain acyl-CoA synthetase *(*ACSL*) *1*, *ACSL4*, and *diacylglycerol acyltransferase 2 *(*DGAT2*), due to the important functions of the genes in fatty acid metabolism in cattle. For single nucleotide polymorphism (SNP) discovery purposes, the coding regions of these six genes were sequenced. Statistical analysis was performed to investigate associations with polymorphisms identified and fatty acid composition.

## Results

### Polymorphism identification and genotyping

We sequenced the full length of coding sequence for the six genes (*FABP4*, *LXRα*, *Cyt b*_5_, *ACSL1*, *ACSL4 *and *DGAT2*) from genome DNA or cDNA of eight animals, including five Japanese Black cattle and three Holstein cattle (primer sequences listed in Table 1). In *FABP4*, sequence comparison among the eight animals revealed four substitutions (220 bpA/G, 328 bpG/A, 348 bpG/C and 396 bpA/G, with the translation initiation site assigned as +1). Two of them were predicted to cause amino acid substitutions, isoleucine to valine at 220 bpA/G (I74V) and valine to methionine at 328 bpG/A (V110M). In *LXRα*, sequence comparison revealed four substitutions (45 bpT/C, 152 bpG/A, 397 bpG/A and 1,314 bpG/T). Two were predicted to cause amino acid substitutions, glycine to glutamic acid at 152 bpG/A (G51E) and valine to isoleucine at 397 bpG/A (V133I). Three synonymous substitutions were detected in *Cyt b*_5 _(195 bpT/C, 201 bpA/G and 360 bpG/A) and *ACSL1 *(282 bpC/T, 516 bpC/G and 1,938 bpT/G). No nucleotide substitution was identified in *ACSL4 *and *DGAT2*.

**Table 1:** Oligonucleotide primers used in this study

Gene	GenBank Accession	Sequence (5' to 3')	Region (Exon)	Amplicon size (bp)	Annealing temp (°C)	Identified SNPs
*FABP4*	NC_007312	FW: CTGGACTCAGAGGGTAATAGCATC RV: TCCAGAGTGAAAAAGAACCATAGGC	1	233	55	
		FW: CACACACACACCTGCTCTTTCTTAG RV: AAAGGTAACATGGTTCAGATCTAGC	2*	397	57	220 bpA/G (I74V)
		FW: CCATGTTACCTTTGATATTTAGCTG RV: ACAACGTATCCAGCAGAAAGTCATG	3–4*	1144	59	328 bpG/A (V110M), 348 bpG/C, 396 bpA/G
*LXRα*	NC_007313	FW: GCACGGTAGCCAAGACATGAGAGTT RV: GGAGTGAAGAGCCTAAGACAGATGC	2–3	1273	60	45 bpT/C, 152 bpG/A (G51E)
		FW: CTAGGACAGCGTGGCGTATGAGAGC RV: ATCTTGGAAAAACAGGTGGGTCTT	4–7	1716	58	397 bpG/A (V133I)
		FW: CAGTGCAGGAGACCCAGCTTCAATC RV: TCATCTGGCTCCACCTCTAG	8–10	1060	55	1,314 bpG/T
		FW: AGGAGTCCAGCACGCCGCAGTCCACT RV: GGAGTGAAGAGCCTAAGACAGATGC	3*	263	62	
		FW: CTAGGACAGCGTGGCGTATGAGAGC RV: TCCTCTTGCCGCTTCATTTTCTTCA	4*	846	60	
*Cyt b*_5_	NC_007325	FW: GAATGTCCCAGGACGAGTTCGCA RV: TCAGTTGAAGTTAAGAGTGGTGGCT	1–4	575	57	195 bpT/C, 201 bpA/G, 360 bpG/A
*ACSL4*	NC_007331	FW: GTCATAGACATCCCTGGAGCAGACA RV: CAGACAGTTCAATGCGAGGCTT	2–15	2020	60	
		FW: CCTTGGGGAGT TTGTCACAGCAT RV: AACTGTTAGAGTGAGAGATGCTGTA	2	771	60	
*ACSL1*	NC_007328	FW: CCAGGATGATGCAAGCCCACG A RV: CTCGTTTTGGAAATCATGGATCACC	2–19	2278	56	282 bpC/T, 516 bpC/G, 1,938 bpT/G
		FW: CCTTGGGGAGTTTGTCACAGCAT RV: CCTGTCTGGGGAGCACATTTCAG	2	439	58	
*DGAT2*	NC_007313	FW: GACCTGTACTGGCTTCGTCATGA RV: AAACGATTTGCAGATGGTTCCTC	1–8	1154	58	

* Primers used for genotyping

Subsequently, we genotyped four polymorphic sites corresponding to the nonsynonymous substitutions identified in *FABP4 *and *LXRα *using 234 Japanese Black cattle. The major allele frequencies were 0.59 (I74V) and 0.71 (V110M) in *FABP4*, and 0.76 (G51E) and 0.86 (V133I) in *LXRα*.

### Effect of the polymorphisms of FABP4 and LXRα on the characteristics of dressed carcasses and fatty acid composition

Effects of the four genotypes of *FABP4 *and *LXRα *on phenotypic traits (fatty acid composition and carcass traits listed in Table 2) were investigated by analysis of variance (ANOVA) (Table 2). Year of testing and sex had significant effects on the characteristics of dressed carcasses and fatty acid compositions as described in previous study [9]. *FABP4 *I74V had a highly significant effect on the percentage of C16:1 (P = 0.0086). *LXRα *V133I had a significant effect on the percentage of C18:2 (P = 0.0121), while no significant effect was found with the polymorphisms *FABP4 *V110M and *LXRα *G51E. These four substitutions did not significantly affect the seven carcass traits analyzed in this study.

**Table 2:** ANOVA on carcass traits and fatty acid composition in Japanese Black population

	n = 234							
	
	Mean	SD	Year	Sex	I74V	V110M	G51E	V133I
*Carcass traits*								

Dressed carcass weight (kg)	437.48	56.23	*	***	ns	ns	ns	ns
Rib-eye area (cm^2^)	55.91	8.22	*	*	ns	ns	ns	ns
Rib thickness (cm)	7.78	0.94	*	*	ns	ns	ns	ns
Subcutaneous fat thickness (cm)	2.97	0.81	ns	ns	ns	ns	ns	ns
Yield estimate (%)	73.74	1.44	ns	ns	ns	ns	ns	ns
BMS	5.96	2.12	ns	ns	ns	ns	ns	ns
BCS	3.93	0.75	**	*	ns	ns	ns	ns
								
*Fatty acid composition *(%)								

C14:0	2.64	0.60	***	ns	ns	ns	ns	ns
C14:1	0.87	0.32	***	ns	ns	ns	ns	ns
C16:0	27.00	2.09	***	***	ns	ns	ns	ns
C16:1	3.82	0.79	***	***	**	ns	ns	ns
C18:0	11.51	1.75	*	**	ns	ns	ns	ns
C18:1	52.01	2.90	**	***	ns	ns	ns	ns
C18:2	2.14	0.55	***	ns	ns	ns	ns	*
MUFA	56.70	3.10	ns	***	ns	ns	ns	ns
SFA	41.15	3.21	*	***	ns	ns	ns	ns
MUFA/SFA	1.39	0.18	*	***	ns	ns	ns	ns
C14:1/(C14:0+C14:1)	24.48	5.09	***	**	ns	ns	ns	ns
C16:1/(C16:0+C16:1)	12.42	2.53	***	***	*	ns	ns	ns
C18:1/(C18:0+C18:1)	81.87	2.76	*	***	ns	ns	ns	ns
Elongation Index	67.32	2.34	*	*	ns	ns	ns	ns

*p < 0.05, **p < 0.01, ***p < 0.001. ns = not significant.

SFA, saturated fatty acid. MUFA, monounsaturated fatty acid. Elongation index, (C16:1+C18:1/C16:0+C16:1+C18:0+C18:1) × 100. BMS, Beef marbling standard. BCS, Beef color standard.

Tukey-Kramer's honestly significant difference (HSD) test was conducted to investigate the detailed effects of *FABP4 *I74V and *LXRα *V133I on fatty acid composition. Table 3 presents the means of each fatty acid and index proportion among genotypes. In *FABP4 *I74V, I/I homozygote exhibited a significantly higher percentage of C16:1 than V/V homozygote, reflecting a high proportion of C16:1/C16:0+C16:1. V/I heterozygote in *LXRα *V133I exhibited a significantly higher percentage of C18:2 than V/V homozygote.

**Table 3:** Effect of *FABP4 *I74V and *LXRα *V133I on fatty acid composition in Japanese Black

	I74V of *FABP4*			V133I of *LXRα*		
	
	I/I n = 82	I/V n = 113	V/V n = 39	V/V n = 174	V/I n = 54	I/I n = 6
Fatty acid composition (%)						
C14:0	2.63	2.47	2.43	2.60	2.53	2.39
C14:1	0.76	0.73	0.68	0.78	0.77	0.62
C16:0	26.96	26.51	26.68	27.02	26.44	26.69
C16:1	3.76^a^	3.56^ab^	3.25^b^	3.65	3.67	3.25
C18:0	12.02	12.20	12.54	12.09	11.89	12.77
C18:1	51.93	52.44	52.27	51.77	52.39	52.49
C18:2	1.95	2.10	2.14	2.08^b^	2.30^a^	1.79^ab^
MUFA	56.45	56.73	56.21	56.20	56.83	56.36
SFA	41.61	41.17	41.66	41.72	40.87	41.85
MUFA/SFA	136.60	139.17	135.78	135.98	140.28	135.29
C14:1/(C14:0+C14:1)	22.35	22.82	21.93	22.68	23.07	21.35
C16:1/(C16:0+C16:1)	12.23^a^	11.88^ab^	10.95^b^	11.94	12.25	10.87
C18:1/(C18:0+C18:1)	81.19	81.11	80.64	81.05	81.48	80.40
Elongation Index	67.54	68.25	68.42	67.55	68.09	68.57

Values are expressed by least squares estimates.

^a, b ^Means with different superscripts within same trait and gene differ significantly at P < 0.05 (Tukey's HSD analysis).

*Fatty acid compositions are calculated in percentages to total of fatty acids or each denominator except MUFA/SFA.

Elongation index, (C18:0+C18:1/C16:0+C16:1+C18:0+C18:1).

We performed Hardy-Weinberg equilibrium test for two SNPs, *FABP4 *I74V and *LXRα *V133I, which revealed significant effects on fatty acid composition. The expected and observed heterozygosity of I74V exhibited 0.484 and 0.483, while those of V133I exhibited 0.243 and 0.231, respectively. No significant differences were detected in two SNPs by Hardy-Weinberg equilibrium test.

## Discussion

Cyt b_5 _is the electron carrier, participating in a series of electron-transfer processes in biological systems, including fatty acid desaturation [10]. ACSL plays a key role in the metabolism of fatty acids. Mercade *et al*. [11] reported the association of the porcine *ACSL4 *polymorphism with growth and percentage of oleic fatty acid. DGAT is the enzyme that catalyzes the final reaction in the synthesis of triglycerides. DGAT2 and SCD1 proteins are located very close to each other in the endoplasmic reticulum, which is a very important criterion for the channeling of substrate [12]. Although these genes relate to fat metabolism, no amino acid substitutions were detected in this study.

In the other two genes, four amino acid substitutions, I74V and V110M in *FABP4 *and G51E and V133I in *LXRα*, were observed. FABP4 is one of cytoplasmic protein family involved in intracellular free fatty acid transport and metabolism, and binds long-chain fatty acids with high affinities. Fatty acid trafficking during lipolysis is mediated by FABP4, and the complex with hormone-sensitive lipase is the first step in an organized lipid-transfer process [13]. LXRα is a transcription factor regulating genes involved in cholesterol and lipid metabolism. LXR directly activates SREBP-1 gene transcription, and the SREBP-1 subsequently activates lipogenic genes such as FASN and SCD-1 [14,15]. From these points of view, the amino acid polymorphisms, found in the current study, may affect the function and be associated with fat-related traits.

We genotyped these four SNPs (I74V and V110M in *FABP4 *and G51E and V133I in *LXRα*) in 234 Japanese Black cattle and investigated the effect on fatty acid composition and carcass traits. ANOVA on carcass traits and fatty acid composition revealed some significance in year and sex effects. Steers had higher dressed carcass weight, rib-eye area, rib thickness and saturated fatty acid proportion, while heifers had higher BCS (darker color) and MUFA proportion. Our results were consistent with previous study described by Zembayashi *et al*. [5] in terms of higher MUFA and lower SFA in heifers. We demonstrated the significant effect of *FABP4 *I74V on palmitoleic acid (C16:1) and *LXRα *V133I on linoleic acid (C18:2). *FABP4 *I74V had a significant effect on C16:1 proportion; the I/I homozygote exhibited 0.5% higher percentage than V/V homozygote (Table 3). Westerling and Hedrick [16] suggested a negative correlation between C16:1 and beef flavor or beef tenderness, so the polymorphism of *FABP4 *I74V might be a candidate marker in beef flavor and tenderness indices. The V/I heterozygote of *LXRα *V133I was 0.2% higher than V/V homozygote, and I/I homozygote was the lowest. Since I/I homozygote was observed in only six cattle (Table 3), we are unable to draw conclusions from such limited numbers. In order to confirm the effect, further investigations would be required in other cattle populations that include adequate frequency of allele I.

FABP4 has been reported as a possible candidate gene for obesity since it was located within a quantitative trait loci (QTL) region contributing to the serum leptin levels in rat [17]. Leptin is a serum protein secreted from adipocytes and is involved in regulating body fat by inhibiting food intake and stimulating energy expenditure. This suggests that FABP4 play an important role in lipid metabolism and homeostasis in adipocyte of cattle.

Michal *et al*. [18] reported that bovine FABP4 genotype significantly affected the marbling score and subcutaneous fat depth in a Wagyu × Limousine F2 population. However, no FABP4 effects on any carcass traits including BMS and subcutaneous fat thickness were found in our study. Since the polymorphism found by Michal *et al*. [18] was detected in 3' untranslated region, the polymorphism might have different phenotypic effects than *FABP4 *I74V. Cho *et al*. [19] identified polymorphisms of *FABP4 *I74V, V110M and 348 bpG/C. The authors showed a putative association with *FABP4 *I74V and back fat thickness in Korean native cattle. Our study shows no such associations in Japanese Black cattle. The reasons may be due to differences of cattle breeds and/or standard for the measurement of BMS and fat thickness.

In human, *LXRα *polymorphism in exon 5 was associated with obesity phenotypes, and relative *LXRα *mRNA expression level was higher in obese women [20]. Yu *et al*. [21] identified two polymorphisms influencing lean and fat growth in pig. The SNP identified in intron 8 was significantly associated with loin eye area and total lipid. Significant associations were also found between the polymorphism in exon 2 and boneless loin and marbling score. Since polymorphisms identified in our study altered amino acid changes but did not affect carcass traits, further work will be necessary to investigate the association between expression level of *LXRα *and carcass traits or fatty acid composition in cattle.

Homology searches for *FABP4 *I74V and *LXRα *V133I among several mammalian spices indicates that the amino acid changes are conservative. The FABP4 protein consists of two α helix domain and 10 β strands domains [22] and the *FABP4 *I74V is located on fifth β sheets. The *LXRα *V133I is located on Zinc finger DNA binding domain [23]. These amino acid replacements may affect structure and therefore function of these proteins.

In this study, our results suggest that *FABP4 *I74V and *LXRα *V133I are in part associated with fatty acid composition. Since the fatty acid composition is a polygenic trait and QTL analysis has shown that the fatty acid composition of livestock species is regulated by many potential candidate genes [24,25], the effect of each gene may be rather small. Additionally, differential expression of adipogenic factors has been known to play a key role in regulating multiple responsive pathways processing fat development and lipid metabolism in cattle adipocytes [26]. Further analysis investigating the interaction of the genetic factors in the biochemical pathway with respect to lipid synthesis and fatty acid metabolism will bring new insight into the fat-related carcass traits in beef cattle. To evaluate the intimate effects of *FABP4 *I74V and *LXRα *V133I, additional analysis using different field population is needed to confirm the effects of these two polymorphisms in the future.

## Conclusion

In summary, we have identified two nonsynonymous substitutions, *FABP4 *I74V and *LXRα *V133I, that were significantly associated with C16:1 (*FABP4 *I74V) and C18:2 content (*LXRα *V133I) in intramuscular fat. In particular, the effect of *FABP4 *I74V might be a good DNA marker for beef quality.

## Methods

### Animals and fatty acid analysis

For genomic DNA and RNA extraction, *Musculaus (M.) trapezius *muscle and subcutaneous adipose tissue around the right neck were collected from five Japanese Black and three Holstein cattle and stored at -80°C until RNA preparation. We extracted genomic DNA from the muscle sample (*M. trapezius*) and RNA from the adipose tissue.

In order to investigate the association between the genotypes and fatty acid composition, we collected muscle samples (*M. trapezius*) of 234 Japanese Black cattle, 141 steers and 93 heifers, as part of the progeny testing performed by the Wagyu Registry Association of Japan from 2002 to 2006. The average age in months at slaughter was 28.5 ± 1.4 (mean ± standard deviation). The intramuscular adipose tissue samples were collected from intramuscular fat of *M. longissimus dorsi *to analyze fatty acid composition. The measurements of fatty acid profile were carried out according to a previous study [7]. Analyzed fatty acid methyl esters were C14:0, C14:1, C16:0, C16:1, C18:0, C18:1 and C18:2 and the composition of each fatty acid was expressed as a percentage. C20:0 and greater sized fatty acids were not included in this analysis since the percentages were lower than 0.5%.

### DNA polymorphism identification

Total RNA extraction from fat tissues and cDNA synthesis were performed according to our previous study [6]. Genomic DNA samples were isolated by proteinase K digestion followed by phenol extraction. Nucleotide sequence of the coding sequence for six genes was identified using cDNA or genomic DNA from five Japanese Black and three Holstein. Sequencing for *Cyt b*_5_, *ACSL1*, *ACSL4 *and *DGAT2 *genes were performed using cDNA, while genomic DNA was used for sequencing in *FABP4 *and *LXRα*.

Primer sets for the PCR amplification and sequencing analysis were designed based on GenBank sequences [*FABP4*: GenBank:NC007312, *LXRα*: GenBank:NC007313, *Cyt b*_5_: GenBank:NC007325, *ACSL1*: GenBank:NC007328, *ACSL4*: GenBank:NC007331 and *DGAT2*: GenBank:NC_007313]. The primer information is listed in Table 1.

After purification of PCR product using a GENCLEAN^® ^II KIT (QBIOgene, CA, USA), standard double-strand DNA cycle sequencing was performed with approximately 20 ng of amplified product using BigDye^® ^Terminator v3.1 Cycle Sequencing Kit (Applied Biosystems, CA, USA) on ABI PRISM^® ^3100 Genetic Analyzer (Applied Biosystems, CA, USA).

### Genotyping

We applied PCR-RFLP methods to genotype four nucleotide substitutions in bovine *FABP4 *and *LXR*α genes. Four PCR primer sets (Table 1) were designed for DNA fragments flanking I74V and V110M in *FABP4*, and G51E and V133I in *LXRα*. The PCR amplification was performed with 20 ng of genomic DNA and TaKaRaEx Taq™ polymerase (TaKaRa, Kyoto, Japan). Amplification was performed with a thermal cycler, GeneAmp PCR System 9700 (Applied Biosystems, CA), with the following thermo-cycling protocol: initial denaturation at 94°C for 2 min, followed by 30 cycles of 94°C for 30 s, 57–62°C for 30 s, and 72°C for 1 min, with a final extension step, 72°C for 7 min. The fragment flanking I74V and V110M in *FABP4 *were digestible with *Nmu*CI and *Nla*III, respectively, and the fragment flanking *LXRα *V133I was digestible with *Hpy*CH4IV. Mismatch PCR-RFLP was also adopted to introduce a *Bse*NI recognition site for the fragment flanking *LXRα *G51E since no suitable restriction enzyme was detected in the site. The digestions were performed in a total of 20 μl volume reaction mixture with approximately 5 μg of PCR products and 3 units of each restriction enzyme. The reaction was incubated at the proper temperature for each restriction enzyme. The digested PCR products were confirmed with undigested products and sequenced homozygous and heterozygous samples by agarose gel electrophoresis.

### Statistical analysis

Fatty acid composition of intramuscular fat and the character of dressed carcasses (the weight of dressed carcass, rib-eye area, rib thickness, subcutaneous fat thickness, yield estimate, beef marbling standard and beef color standard) were tested statistically by ANOVA with a model that accounted for year of testing, sex, four genotypes without interaction. Tukey's HSD test was performed for further analysis. Genotypic distributions and allele frequencies were calculated and Hardy-Weinberg equilibrium test was performed using Arlequin version 3.1 [27].

## Authors' contributions

TH carried out the genotyping. AT carried out the fatty acid analysis. HO carried out the genotyping. SS participated in the design and coordination of the study. MT helped to draft the manuscript. KO carried out the statistical analysis. FM conceived of the study, and participated in its design and coordination. HM conceived of the study, and participated in its design and coordination and helped to draft the manuscript. All authors read and approved the final manuscript.
